# Modeling of nitrogen solubility in normal alkanes using machine learning methods compared with cubic and PC-SAFT equations of state

**DOI:** 10.1038/s41598-021-03643-8

**Published:** 2021-12-22

**Authors:** Seyed Ali Madani, Mohammad-Reza Mohammadi, Saeid Atashrouz, Ali Abedi, Abdolhossein Hemmati-Sarapardeh, Ahmad Mohaddespour

**Affiliations:** 1 Department of Chemical and Petroleum Engineering, Sharif University of Technology, Tehran, Iran; 2 Department of Petroleum Engineering, Shahid Bahonar University of Kerman, Kerman, Iran; 3 Department of Chemical Engineering, Amirkabir University of Technology (Tehran Polytechnic), Tehran, Iran; 4 College of Engineering and Technology, American University of the Middle East, Egaila, Kuwait; 5 College of Construction Engineering, Jilin University, Changchun, 130012 China; 6 Key Laboratory of Continental Shale Hydrocarbon Accumulation and Efficient Development, Ministry of Education, Northeast Petroleum University, Daqing, 163318 China

**Keywords:** Energy science and technology, Engineering, Mathematics and computing

## Abstract

Accurate prediction of the solubility of gases in hydrocarbons is a crucial factor in designing enhanced oil recovery (EOR) operations by gas injection as well as separation, and chemical reaction processes in a petroleum refinery. In this work, nitrogen (N_2_) solubility in normal alkanes as the major constituents of crude oil was modeled using five representative machine learning (ML) models namely gradient boosting with categorical features support (CatBoost), random forest, light gradient boosting machine (LightGBM), k-nearest neighbors (k-NN), and extreme gradient boosting (XGBoost). A large solubility databank containing 1982 data points was utilized to establish the models for predicting N_2_ solubility in normal alkanes as a function of pressure, temperature, and molecular weight of normal alkanes over broad ranges of operating pressure (0.0212–69.12 MPa) and temperature (91–703 K). The molecular weight range of normal alkanes was from 16 to 507 g/mol. Also, five equations of state (EOSs) including Redlich–Kwong (RK), Soave–Redlich–Kwong (SRK), Zudkevitch–Joffe (ZJ), Peng–Robinson (PR), and perturbed-chain statistical associating fluid theory (PC-SAFT) were used comparatively with the ML models to estimate N_2_ solubility in normal alkanes. Results revealed that the CatBoost model is the most precise model in this work with a root mean square error of 0.0147 and coefficient of determination of 0.9943. ZJ EOS also provided the best estimates for the N_2_ solubility in normal alkanes among the EOSs. Lastly, the results of relevancy factor analysis indicated that pressure has the greatest influence on N_2_ solubility in normal alkanes and the N_2_ solubility increases with increasing the molecular weight of normal alkanes.

## Introduction

Gas and fluids interactions are an undeniable part of many industrial procedures, which plays some major roles in many industries like petrochemical^1–3^, oil and gas^4–9^, medicine^10^, food^11,12^, environment^13,14^, polymer^15,16^, etc. Among the common gaseous phases normally present in the mentioned environments, colorless odorless nitrogen (N_2_) is one of the most common gases included as the feed or product in many processes. On the other hand, the presence of this gas as the dominant part of atmosphere components makes it an important case to be investigated accurately. The oil and gas industry would not be an exception, and N_2_ applications are observed in many subsidiaries of this industry, from the upstream to downstream. As a clear example, N_2_ and its related treatments have been used since few decades ago because of its unique properties for enhanced oil recovery (EOR) operations^17–19^. Usually, carbon dioxide (CO_2_) or N_2_ gases are continuously injected into the oil reservoir for miscible/immiscible oil displacement. These gases are extracted back out with the recovered oil, recaptured, and reinjected along with new gas until as much oil as possible is produced^20^. Cost efficiency and higher feasibility make some advantages for this component (N_2_) in comparison with CO_2_ and methane (CH_4_)^21,22^. However, N_2_ has been commonly utilized in deep reservoirs as it needs a higher injection pressure to gain miscibility with the reservoir fluids than does CO_2_^20^. Also, in the midstream, N_2_ is used in pipeline drying, which is an essential part of pipeline commissioning to prevent unwanted aerosols through contaminant displacing^23^. There are many significant instances of N_2_ usage in downstream, like nitrogen purging which is a technique to avoid unintentional reaction of hazardous gas and hydrocarbons through the oxygen reduction in the environments that is susceptible to explosion^24^ that is a similar technique which is used in nitrogen blanketing^25^ in hydrocarbon storage tanks. Crude oil is a complex mixture of hydrocarbons. Achieving reliable predictions for the thermodynamics and phase equilibrium data of N_2_/oil systems is complex and difficult. Alkanes are the major constituents of crude oil and most petroleum products. Therefore, in many studies, the behavior of alkanes and the desired gas like N_2_ is studied first, and the obtained information will be later generalized to crude oil.

Solubility is one of the most important thermodynamics values representing the value of a gas dissolution in a liquid at a specific pressure and temperature. While many analytical methods are used to calculate the solubilities of gases in liquids mainly through the equations of state (EOSs)^26–29^, the accuracy of their prediction, especially in some critical industrial applications, has been a serious challenge yet. Based on previous experiments, the solubility of N_2_ in hydrocarbons is positively affected by increasing pressure and temperature^26–28^. Furthermore, as the molecular weight rises, N_2_ solubility increases, as evidenced by laboratory experiments^29^. Properly estimating phase equilibrium data in binary systems containing N_2_ and a hydrocarbon is difficult. Because, based on the classification scheme of Van Konynenburg and Scott^30,31^, binary systems of a hydrocarbon and N_2_ are recognized as type III phase diagrams, except the binary system of N_2_ + CH_4_, which is recognized as a type I system^30,31^. Risk of energy waste and potential hazards exist in operations which use N_2_. As a result, solubility data is critical for predicting an appropriate quantity of N_2_ to use in this operation, and it can improve plant safety. Studies with heavy hydrocarbons are particularly challenging due to their complexity. Furthermore, the dangers of high-temperature and/or high-pressure conditions in industrial operations make the extensive experiments an undesirable option. As a result, modelling with experimental data would be an alternative.

Mainly, the strategies for the prediction of N_2_ solubility in hydrocarbon solvents or petroleum blends rely on experimental and semi-empirical models like EOSs, and are comparable to those utilized to estimate the solubility of other gasses like CH_4_, CO_2_, and hydrogen^32–37^. In compressed N_2_, the vapor-phase solubilities of n-Decane, ferf-butylbenzene, 2,2,5-trimethylhexane, and n-dodecane were determined by Davila et al.^38^ and the second virial cross coefficients ($B_{12}$) were computed using these data^38^. A static equilibrium cell was used by Tong et al.^29^ to test the solubilities of N_2_ in four n-paraffin hydrocarbons (Decane, Eicosane, Octacosane, and Hexatriacontane). The Soave–Redlich–Kwong (SRK) and Peng-Robinson (PR) EOS were applied to analyze the data. The results show a growing trend in N_2_ solubility with rising pressure, temperature, and n-paraffin chain length^29^. N_2_ solubilities in various naphthenic (trans-Decalin and cyclohexane) and aromatic (naphthalene, 1-methylnaphthalene, benzene, phenanthrene, pyrene) solvents were determined by Gao et al.^26^ using a static cell. When a single interaction parameter ($C_{ij}$) is employed in each binary system, the PR-EOS was demonstrated to fit the model^26^. Privat et al.^39,40^ used the PR EOS combined with the group contribution method, called the PPR78 model, for predicting phase equilibrium data of mixtures containing various hydrocarbons and N2. This model is able to predict temperature-dependent binary interaction parameters (kij). The mentioned model provided satisfying results with an overall deviation lower than 10%. They also mentioned that for the hydrocarbon + N_2_ systems (except CH_4_); k_ij_ is a decreasing function of temperature^39,40^. At low temperatures, Justo-Garcia et al.^41^ modeled vapor–liquid-liquid equilibria (VLE) for N_2_ and alkanes in three distinct ternary systems. The findings demonstrate that both SRK and PC-SAFT EOSs estimate the experimentally observed values with reasonable accuracy^41^. In another study, Justo-Garcia et al.^42^ used the SRK and PC-SAFT EOSs to model three-phase vapor–liquid–liquid equilibria for a combination of natural gas having high N_2_ content. The results revealed that the PC-SAFT EOS accurately predicts phase behavior, but the SRK EOS suggests a three-phase region that is larger than what was observed experimentally^42^. The Krichevsky–Ilinskaya equation was used by Zirrahi et al.^27^ to estimate the solubility of light solvents (CO_2_, N_2_, CH_4_, C_2_H_6_, and CO) in bitumens from five Alberta reservoirs. The gas phase is analyzed applying the PR-EOS. The suggested model is then validated using experimental data on light solvent solubility. The results demonstrated that the proposed model accurately reflects known solubility data in bitumen for light hydrocarbons (CH_4_ and C_2_H_6_) and non-hydrocarbon solvents (N_2_, CO_2_, and CO)^27^. Haghbakhsh et al.^43^ investigated the vapor–liquid equilibria of binary N_2_–hydrocarbon mixtures across an extensive range of temperature and pressure applying PR and ER EOSs. They introduced a new correlative mode for the proposed equations to improve accuracy, which was likely to be effective, improving accuracy by up to three times^43^. Thermo-physical characteristics of CO_2_ and N_2_/bitumen solutions were studied by Haddadnia et al.^28^. Furthermore, PR-EOS was used to describe the calculated solubility^28^. PC-SAFT and SRK EOSs were employed by Wu et al.^44^ to estimate gas solubilities in n-alkanes. The PC-SAFT EOS was found to be able to accurately predict an empirically observed linear connection between gas solubilities in n-alkanes and their carbon number. Despite its satisfactory accuracy for gas solubility in lighter n-alkanes, the SRK EOS typically produces significantly poorer results than the PC-SAFT EOS^44^. Tsuji et al.^45^ investigated N_2_ and oxygen gas solubilities in benzene, divinylbenzene, and styrene. For a particular isotherm, gas solubility in liquids had a linear pressure dependency and declined with rising temperature. Ultimately, PR-EOS was implemented to predict gas solubilities^45^. Aguilar-Cisneros et al.^46^ determined the solubility of N_2_, CO_2_, and CH_4_ in petroleum fluids using the PR-EOS in conjunction with various mixing rules in systems including bitumens, heavy oils, refinery cuts, and coal liquids. The universal and van der Waals mixing rules revealed satisfactory outcome between experimental data and predicted values, while the modified Huron-Vidal of order one mixing rule produced large discrepancies^46^.

During the last decade, alongside the developments of intelligent methods based on machine learning (ML) techniques, many attempts have been made to predict thermodynamic results with a higher accuracy based on reliable experimental data. Abdi-Khanghah et al.^47^ studied alkane solubility in supercritical CO_2_. Two kinds of artificial neural networks were used for their study: Radial basis function (RBF) and multi-layer perceptron (MLP) artificial neural network (ANN). The MLP-ANN outperformed the RBF-ANN in predicting n-alkane solubility in supercritical CO_2_^47^. Songolzadeh et al.^48^ demonstrated that the PSO–LSSVM model is an effective technique for predicting n-alkane solubility in supercritical CO_2_ with high accuracy. The least-squares support vector machine (LSSVM) was employed, which was tuned using two different optimizing algorithms: particle swarm optimization (PSO) and cross-validation-assisted Simplex algorithm (CV-Simplex)^48^. Chakraborty et al.^49^ developed a set of data-driven models capable of predicting VLE for the binary systems of C_10_-N_2_ and C_12_-N_2_. In comparison to the VLE modeled using the PR-EOS, both models significantly improved the estimated value of binary mixture equilibrium pressure^49^. Mohammadi et al.^50^ implemented different ML models to predict hydrogen solubility in various pure hydrocarbons in wide pressure and temperature ranges and compared them with some of the common EOSs. Their results showed that using intelligent models shows more precise results than the common usage of EOSs in hydrogen solubility estimation^50^. To predict nitrogen solubility in unsaturated, cyclic and aromatic hydrocarbons, Mohammadi et al.^51^ employed a convolutional neural network (CNN) and the results showed that pressure is the most significant factor for nitrogen solubility in unsaturated hydrocarbons. In general, prediction based on EOSs semi-analytical methods has been the common way to estimate the N_2_ solubilities in alkanes. On the other hand, the mentioned method is case-specific and it is limited to some defined hydrocarbons with specific parameters for each EOS. Hence, using intelligent models like proper ML algorithms and reliable experimental data may lead to a model for predicting N_2_ solubility in normal alkanes with high accuracy and this helps to accelerate predictions.

In this study, we use a dataset containing 1982 experimental N_2_ solubility data points for 19 distinct normal alkanes gathered under various operating states. Models for estimating N_2_ solubility in normal alkanes are constructed using well-known ML algorithms namely k-nearest neighbor (k-NN) and random forest (RF), as well as innovative ML methods such as extreme gradient boosting (XGBoost), gradient boosting with categorical features support (CatBoost), and light gradient boosting machine (LightGBM). Furthermore, statistical parameters and graphical error assessments are used to verify the validity of the suggested models. Numerous N_2_ solubility systems are predicted by the methods proposed in this research and five EOSs, namely perturbed-chain statistical associating fluid theory (PC-SAFT), Redlich-Kwong (RK), Peng-Robinson (PR), Soave–Redlich–Kwong (SRK), and Zudkevitch-Joffee (ZJ). Eventually, the relevancy factor is utilized to assess the relative impact of input parameters on N_2_ solubility in normal alkanes.

## Data collection

The modeling of N_2_ solubility in normal alkanes was performed using a large solubility databank containing 1982 data points collected from the literature^29,52–91^. The properties of 19 normal alkanes (nC_1_ to nC_36_) utilized in this survey are presented in Table 1.

**Table 1:** The normal alkanes utilized in this survey.

Solvent	Carbon number	T_c_ (K)	P_c_ (MPa)	Mw (g/mol)
Methane	1	190.56	4.599	16.043
Ethane	2	305.32	4.872	30.07
Propane	3	369.83	4.248	44.1
Butane	4	425.12	3.796	58.12
n-Pentane	5	469.7	3.37	72.15
n-Hexane	6	507.6	3.025	86.18
n-Heptane	7	540.2	2.74	100.2
n-Octane	8	568.7	2.49	114.23
n-Nonane	9	594.6	2.29	128.25
n-Decane	10	617.7	2.11	142.28
Undecane	11	639	1.98	156.31
n-Dodecane	12	658	1.82	170.33
Tridecane	13	675	1.68	184.36
Tetradecane	14	693	1.57	198.39
Pentadecane	15	708	1.48	212.41
n-Hexadecane	16	723	1.4	226.44
n-Eicosane	20	768	1.07	282.5
n-Octacosane	28	832	0.727	394.8
n-Hexatriacontane	36	872	0.47	507

The inputs of the models were chosen to be temperature (K), pressure (MPa), and molecular weight (g/mol) of normal alkanes, whereas N_2_ solubility (in terms of mole fraction) was the desired output. The statistical details of the N_2_ solubility databank used for modeling are tabulated in Table 2. The validity, accuracy, and applicability of the model depend on the quantity and variety of N_2_ solubility data collected in different systems. The broad ranges of pressure (0.0212–69.12 MPa), temperature (91.21–703.4 K), and normal alkanes (nC_1_ to nC_36_) can lead to a reliable general model for estimating the solubilities of N_2_ in normal alkanes.

**Table 2:** The statistical information of the N_2_ solubility databank used in this paper.

	Mw (g/mol)	Temperature (K)	Pressure (MPa)	N_2_ solubility (mole fraction)
Minimum	16.04	91.21	0.0212	0.0008
Maximum	507	703.4	69.12	0.9515
Mean	99.22	336	12.5	0.2203
Std. Deviation	73.88	132.8	13.29	0.1964
Skewness	1.79	− 0.098	1.45	1.136
Kurtosis	6.294	− 0.8567	1.543	0.8351

## Models’ implementation

### Algorithms’ selection

Due to recent advances in computation capacities and also the advent of new machine learning algorithms, there are many choices to use as algorithms for the problem under consideration. Because of the size of the dataset and small instance number and also based on the limited number of the features, some of the non-parametric ML models which mainly focus on the dataset and do not suffer from the small size of the dataset were noticed as the best choices in this case.

### K-nearest neighbors (k-NN)

The k-NN method is an ML technique that is employed to solve both classification and regression problems. This supervised algorithm is widely used as a non-parametric technique for various applications^92^. In this algorithm, the *k* is the number of neighbors which are assigned to a new sample to predict the target based on its inheritance from these *k* samples that are closest to the new sample using a uniform weight assigning system or a specific distance function^93^. Distance function is a tool to allocate a weight to each of the *k* samples features to identify its contribution in final predicted value. Minkowski distance equation is the typical choice for the distance function. The general form of this equation is provided in Eq. (1), where *X* and *Y* are two samples feature sets. This function turns to Manhattan or Euclidean distance function in most of the cases by using the *p* = *1* or *p* = *2*, respectively. Finding and selection of the optimal value of the *k* hyper-parameter is the most crucial stage in the training of this algorithm to achieve a satisfactory accuracy. Hence, the algorithms are run by a wide range of *k* value and the optimal case is revealed based on the comparison of statistical accuracy measurements among the explored cases.

1 $$\begin{aligned} D\left( {X,Y} \right) & = \left( {\mathop \sum \limits_{i = 1}^{n} \left| {x_{i} - y_{i} } \right|^{p} } \right)^{\frac{1}{p}} \\ X & = \left( {x_{1} ,x_{2} , \ldots ,x_{n} } \right) \;{\text{and}}\;Y = \left( {y_{1} ,y_{2} , \ldots , y_{n} } \right) \in {\mathbb{R}}^{n} \\ \end{aligned}$$

### Random forest

Random forest is a bagging supervised learning technique for classification and regression using the ensemble learning approach based on CART (Classification and Regression Trees)^94^. This algorithm avoids high prediction variance, which is a common issue in the decision tree algorithm. Random forests have trees, which run parallelly. These trees do not have any interaction with each other during the forest construction. It works by training a large number of decision trees and then determining the class that is the mean prediction of the individual trees in regression cases. At each node, the number of attributes that may be divided is limited to a certain proportion of the total which is known as the hyperparameter. This guarantees that the ensemble model does not depend too strongly on any specific attribute and that all potentially predictive variables are considered equally. In any CART tree training, the random forest technique picks the training dataset *T*_*i*_, randomly from the complete training set *T*, by replacement (i.e., bootstrapping sampling). The data that was not included in the random sampling technique is referred to as "out-of-bag" data. The random forest technique picks *N* features or input variables randomly from a set of *M* input independent factors (*N* < *M*) while building each CART tree. According to the randomly picked *T*_*i*_ and *M* characteristics, the best splitting for each CART tree is calculated. The final results of the regression are being determined via majority voting. To increase the estimation precision, the averaged prediction reduces the averaged squared error on the individual estimations produced from an individual CART tree. The resulting ensemble trees are designated as follows (Eq. 2):

2 $$\begin{gathered} \left\{ {\phi_{{T_{b} ,m}} \left| {b = 1, \ldots ,B} \right.} \right\} \hfill \\ \hat{Y} = \phi_{T,P} \left( X \right) = \frac{1}{B}\mathop \sum \limits_{b = 1}^{B} \phi_{{T_{b} ,m}} \left( X \right) \hfill \\ \end{gathered}$$

### Extreme gradient boosting (XGBoost)

**Figure 1:**
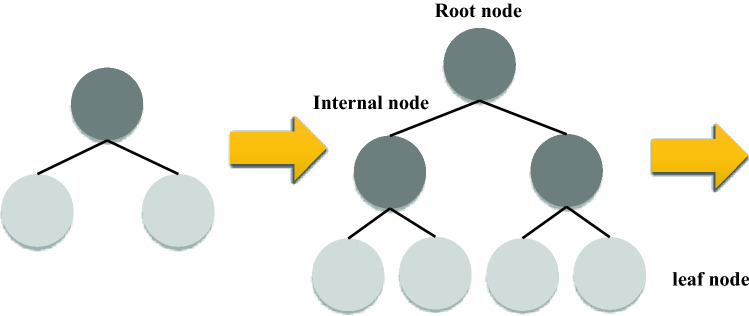
Level-by-level tree development in XGboost.

The fundamental concept behind a tree-based ensemble method is to use an ensemble of classification and regression trees (CARTs) to fit training data using a regularized objective function minimization. One of those other tree-based models is XGBoost, which is part of the gradient boosting decision tree framework (GBDT). To further explain the construction of the CART, each cart is made up of (I) a root node, (II) internal nodes, and (III) leaf nodes, as illustrated in Fig. 1. The root node, which represents the entire dataset, is split into internal nodes by the binary decision technique, whilst the leaf nodes reflect the final classifications. In gradient boosting, a sequence of basic CATRs are created simultaneously, with the weight of each individual CART being adjusted via the training process^95^.

An ensemble of n trees must be trained to predict the *y* for a specific dataset, *m* and *n* respectively show the count of features and instances.

3 $$\begin{aligned} \hat{y}_{i} = \sum\limits_{k = 1}^{N} {f_{k} \left( {X_{i} } \right),\;\;\,f_{k} \in f} \\ & With\;f= \left\{ {f(X) = \omega_{q(x)} } \right\},\;\left( {q:{\mathbb{R}}^{m} \to T,\;\omega \in {\mathbb{R}}^{T} } \right) \\ \end{aligned}$$

where the decision rule $q\left( x \right)$ maps the example to the binary leaf index. $n$ shows the regression trees space, $f_{k}$ shows the *k*th independent tree, *T* represents the count of tree’s leaves, and *w* shows the leaf’s weight in Eqs. 3 and 4.

The minimization of the regularized objective function $L$ is used to determine the ensemble of trees:

4 $$\begin{aligned} & L = \sum\limits_{i}^{n} {l(\hat{y}_{i} ,y_{i} ) + \sum\limits_{k}^{N} {\Omega \left( {f_{k} } \right)} } \\ & With\;\Omega (f) = \gamma T + \frac{1}{2}\lambda \left\| \omega \right\|^{2} \\ \end{aligned}$$

where *Ω* shows the regularization term that helps to reduce overfitting by reducing the model's complexity; *l* stands for a loss function that is differentiable and convex; *γ* is the minimal loss reduction required to split a new leaf; and *λ* displays the regulation coefficient. It is worth noting that in these equations *λ* and *γ* assist to increase model variance and avoid overfitting.

The objective function for each individual leaf is reduced in the gradient boosting technique, and additional branches are added sequentially.

5 $$L^{(t)} = \sum\limits_{i = 1}^{n} {\left\{ {l(y_{i} ,\hat{y}_{i}^{(t - 1)} ) + f_{t} (X_{i} )} \right\}} + \Omega (f_{t} )$$

The *t*-th iteration of the above-mentioned training procedure is represented by *t*. The XGBoost method aggressively adds the space of regression trees to greatly improve the ensemble model, which is sometimes dubbed "greedy algorithm". As a result, the model output is updated continuously by minimizing the objective function:

6 $$\hat{y}_{i}^{(t)} = \hat{y}_{i}^{(t - 1)} + f_{t} (X_{i} )$$

The XGBoost takes use of a shrinkage technique in which newly added weights are scaled by a learning factor rate after each stage of boosting. This minimizes the risk of overfitting by reducing the impact of future additional trees on each available individual tree^96^.

### Light gradient boosting machine (LightGBM)

**Figure 2:**
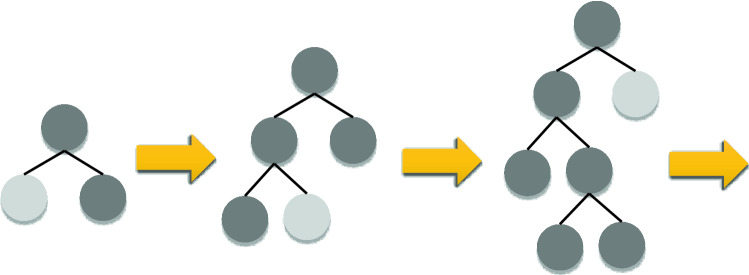
Leaf-wise tree development in LightGBM.

LightGBM is a novel gradient learning framework based on the decision tree concept. The main advantages of LightGBM over XGBoost are that it uses less memory, uses a leaf-wise growth method with depth constraints, and uses a histogram-based technique to speed up the training process. LightGBM discretizes continuous floating-point eigenvalues to *k* bins through using the aforementioned histogram technique, resulting in a *k*-width histogram. Furthermore, the histogram technique does not require additional storing of pre-sorted results, and values may be stored in an 8-bit integer after feature discretization, reducing memory usage to 1/8. Despite this, the model's accuracy suffers as a result of the harsh partitioning method. LightGBM also employs a leaf-by-leaf technique, which is more successful than the usual level-by-level strategy. The reason for this inefficiency in level-wise approach is that at each step, only leaves from the same layer are examined, resulting in unnecessary memory allocation. Alternatively, at each stage of the leaf-wise method, the algorithm finds the leaves with the largest branching gain, and then proceeds to the branching cycle. In comparison to the horizontal direction, errors can be reduced and greater precision can be attained with the same number of segmentations. The leaf-wise tree development technique is illustrated in Fig. 2. The disadvantage of leaf orientation is that it forces you to build deeper decision trees, which invariably leads to overfitting. On the other hand, LightGBM prevents overfitting while maintaining high efficiency by imposing a maximum depth restriction on the leaf top^97,98^.

For a specific training dataset $X = \left\{ {(x_{i} ,y_{i} )} \right\}_{{_{i = 1} }}^{m}$, LightGBM searches an approximation $\hat{f}\left( x \right)$ to the function f*(x) to minimize the expected values of specific loss functions *L (y, f (x))*:

7 $$\hat{f}\left( x \right) = \arg \mathop {\min }\limits_{f} E_{y,x} L(y,f(x))$$

LightGBM ensembles many *T* regression trees $\mathop \sum \limits_{t = 1}^{T} f_{t } \left( x \right)$ to approximate the model. The regression trees are defined as *w*_*q(x)*_, $q \in \left\{ {1, \, 2, \ldots ,N} \right\}$, where *q* shows the decision rule of trees*, N* is defined as the count of tree leaves, and *w* denotes a vector shows the sample weights of leaf nodes. The model is trained in the additive form at step *t*:

8 $$G_{t} \cong \sum\limits_{i = 1}^{N} {L(y_{i} ,F_{t - 1} (x_{i} ) + f_{t} (x_{i} ))}$$

To estimate the objective function, the newton's approach is employed.

### Gradient boosting with categorical features support (CatBoost)

CatBoost, which employs one hot max size (OHMS) that is a permutation technique beside the target-based statistics, employs categorical columns for categorical boosting. For a new split of the present tree, a greedy approach is utilized in this methodology, allowing CatBoost to identify the exponential evolution of the feature combination^99^. In CatBoost, for each feature with more categories than OHMS, the following steps are applied:

1. Records are divided into subsets at random.
2. Integer conversion of labels
3. Convert categorical features to numeric values as follows:
  9 $$avg\;Target = \frac{countInClass + prior}{{totalCount + 1}}$$

where $countInClass$ is the number of targets having a value of one for a category attribute, and $totalCount$ is the number of preceding objects (the starting parameters specify *prior* to count objects)^100,101^.

### Equations of state (EOSs)

EOS is a mathematical expression for the connection among a substance's volume, temperature, and pressure. This equation may be used to explain VLE, volumetric behavior, and thermodynamic properties of mixtures and pure substances. EOSs are used to estimate the phase behavior of petroleum fluids. As previously stated, EOSs have poor predictors of gas solubility in solvents, particularly under complicated working circumstances. Five EOSs were used to assess N_2_ solubility in hydrocarbons in this research, and their reliability in predicting N_2_ solubility is compared to ML algorithms. Mathematical equations of implemented EOSs are shown in Table 3. Table 4 also shows the parameters of the EOSs. Also, some required molecular parameters corresponding to each substance which is investigated with PC-SAFT EOS are provided in Table 5. Besides, a proper mixing rule is needed to use for estimation of each mixture’s parameters. In this study, van der Waals one-fluid mixing rules have been utilized, and its corresponding mathematical expression is provided in Table 4.

**Table 3:** EOSs Formulas utilized in this study.

EOS	Formula	References
ZJ	$P = \frac{RT}{{\nu - b}} - \frac{\alpha }{{T^{1/2} \nu (\nu + b)}}$	^102^
RK	$P = \frac{RT}{{\nu - b}} - \frac{\alpha }{\sqrt T \nu (\nu + b)}$	^103^
SRK	$P = \frac{RT}{{\nu - b}} - \frac{a\alpha }{{(\nu + c)\left( {\nu + b + 2c} \right)}}$	^103,104^
PR	$P = \frac{RT}{{\nu - b}} - \frac{a\alpha }{{(v + c)(v + 2c + b) + (b + c)(v - b)}}$	^103,104^
PC-SAFT	$\tilde{a} = \frac{A}{kTN} = \tilde{a}^{hc} + \tilde{a}^{id} + \tilde{a}^{disp} + \tilde{a}^{assoc}$	^105,106^

**Table 4:** Parameters of EOSs and mixing rules.

EOS	Parameters	References
ZJ	Parameter *α and b* are calculated as functions of temperature and pressure. For complex mixtures,$b_{i} = b_{i}^{ZJ} \left[ {1 + b_{0} \left( {\frac{T}{{T_{C} }} - 1} \right)} \right]$	^102^
RK	$$\begin{gathered} a = {0}{\text{.42748}}\frac{{R^{2} T_{C}^{2.5} }}{{P_{C} }} \hfill \\ b = {0}{\text{.08664}}\frac{{RT_{C} }}{{P_{C} }} \hfill \\ \end{gathered}$$	^103^
SRK	$$\begin{gathered} a = {0}{\text{.42747}}\frac{{\left( {RT_{C} } \right)^{2} }}{{P_{C} }} \hfill \\ b = {0}{\text{.08664}}\frac{{RT_{C} }}{{P_{C} }} \hfill \\ m = 0.48508 + 1.5517\omega - 0.1561\omega^{2} \hfill \\ \alpha = \left[ {1 + m\left( {1 - \sqrt {T_{r} } } \right)} \right]^{2} \hfill \\ c = \frac{{0.40768RT_{C} (0.29441 - Z_{RA} )}}{{P_{C} }} \hfill \\ Z_{RA} = 0.29506 - 0.08775\omega \hfill \\ \end{gathered}$$	^103,104^
PR	$$\begin{gathered} \alpha = \left[ {1 + m\left( {1 - \sqrt {T_{r} } } \right)} \right]^{2} \hfill \\ a = 0.45724\frac{{(RT_{C} )^{2} }}{{P_{C} }} \hfill \\ m = 0.3796 + 1.485\omega - 0.1644\omega^{2} + 0.01667\omega^{3} \hfill \\ b = {0}{\text{.07780}}\frac{{RT_{C} }}{{P_{C} }} \hfill \\ c = \frac{{0.40768RT_{C} (0.29441 - Z_{RA} )}}{{P_{C} }} \hfill \\ {\text{For non - hydrocarbons and hydrocarbons lighter than C}}_{{7}} : \hfill \\ c = \frac{{0.50033RT_{C} }}{{P_{C} }}(0.25969 - Z_{RA} ) \hfill \\ Z_{RA} = 0.29506 - 0.08775\omega \hfill \\ \end{gathered}$$	^103,104^
PC-SAFT	$$\begin{gathered} {\tilde{\text{a}}}^{{{\text{hc}}}} = \overline{m}{\tilde{\text{a}}}^{{{\text{hs}}}} + {\tilde{\text{a}}}^{{{\text{chain}}}} = \overline{m}{\tilde{\text{a}}}^{{{\text{hs}}}} - \sum\limits_{i} {x_{i} } (m_{i} - 1)\ln g_{ij}^{hs} \hfill \\ \overline{m} = \sum\limits_{i} {x_{i} } m_{i} \hfill \\ {\tilde{\text{a}}}^{{{\text{hs}}}} = \frac{1}{{\zeta_{0} }}\left[ {\frac{{3\zeta_{1} \zeta_{2} }}{{1 - \zeta_{3} }} + \frac{{3\zeta_{2}^{3} }}{{\zeta_{3} (1 - \zeta_{3} )^{2} }} + \left( {\frac{{\zeta_{2}^{3} }}{{\zeta_{3}^{2} }} - \zeta_{0} } \right)\ln (1 - \zeta_{3} )} \right] \hfill \\ \zeta_{n} = \frac{\pi }{6}\rho \sum\limits_{i} {x_{i} } m_{i} d_{i}^{n} \, n \in \left\{ {0,1,2,3} \right\}, \, \eta = \zeta_{3} \hfill \\ d_{i} = \sigma_{i} \left[ {1 - 0.12\exp \left( { - 3\frac{{\varepsilon_{i} }}{kT}} \right)} \right] \hfill \\ g_{ij}^{hs} = \frac{1}{{1 - \zeta_{3} }} + \left( {\frac{{d_{i} d_{j} }}{{d_{i} + d_{j} }}} \right)\frac{{2\zeta_{2} }}{{(1 - \zeta_{3} )^{2} }} + \left( {\frac{{d_{i} d_{j} }}{{d_{i} + d_{j} }}} \right)^{2} \frac{{2\zeta_{2}^{2} }}{{(1 - \zeta_{3} )^{2} }} \hfill \\ {\tilde{\text{a}}}^{{{\text{dis}}}} = - 2\pi \rho I_{1} \left( {\eta ,\overline{m}} \right)\overline{{m^{2} \varepsilon \sigma^{3} }} - \pi \rho \overline{m}C_{1} \left( {\eta ,\overline{m}} \right)I_{2} \left( {\eta ,\overline{m}} \right)\overline{{m^{2} \varepsilon^{2} \sigma^{3} }} \hfill \\ I_{1} \left( {\eta ,\overline{m}} \right) = \sum\limits_{i = 0}^{6} {a_{i} } (\overline{m})\eta^{i} { , }I_{2} \left( {\eta ,\overline{m}} \right) = \sum\limits_{i = 0}^{6} {b_{i} } (\overline{m})\eta^{i} \hfill \\ \end{gathered}$$ where *a*_*i*_ and b_i_ depend on the chain length as given in Gross and Sadowski^105^ $$\begin{gathered} C_{1} = \left[ {1 + \overline{m}\frac{{8\eta - 2\eta^{2} }}{{\left( {1 - \eta } \right)^{4} }} + (1 - \overline{m})\frac{{20\eta - 27\eta^{2} + 12\eta^{3} - 2\eta^{4} }}{{\left[ {\left( {1 - \eta } \right)\left( {2 - \eta } \right)} \right]^{2} }}} \right] \hfill \\ \overline{{m^{2} \varepsilon \sigma^{3} }} = \sum\limits_{i} {\sum\limits_{j} {x_{i} x_{j} } } m_{i} m_{j} \left( {\frac{{\varepsilon_{ij} }}{kT}} \right)\sigma_{ij}^{3} \hfill \\ \overline{{m^{2} \varepsilon^{2} \sigma^{3} }} = \sum\limits_{i} {\sum\limits_{j} {x_{i} x_{j} } } m_{i} m_{j} \left( {\frac{{\varepsilon_{ij} }}{kT}} \right)^{2} \sigma_{ij}^{3} \hfill \\ \varepsilon_{ij} = \sqrt {\varepsilon_{i} \varepsilon_{j} } \left( {1 - k_{ij} } \right) \hfill \\ \sigma_{ij} = \frac{{\left( {\sigma_{i} + \sigma_{j} } \right)}}{2} \hfill \\ \end{gathered}$$ The expressions for the contributions from the dispersion and ideal gas are identical to those of Gross and Sadowski^105^	^105,106^
Van der Waals one-fluid mixing rules	$$\begin{gathered} a = \sum\limits_{i = 1}^{N} {\sum\limits_{j = 1}^{N} {z_{i} z_{j} \sqrt {a_{i} a_{j} } } } \left[ {1 - k_{ij} (T)} \right] \hfill \\ b = \sum\limits_{i = 1}^{N} {z_{i} b_{i} } \hfill \\ \end{gathered}$$	^103,107^

**Table 5:** Parameters of PC-SAFT EOS^105,108,109^.

Component	Formula	Molecular weight (Mw) [g/mol]	$$T_{c}$$[K]	$$P_{c}$$[MPa]	Segment number ($$m)$$ [–]	Segment diameter ($$\sigma )$$ [Å]	Energy parameter ($$\varepsilon /k)$$ [K]
Nitrogen	$N_{2}$	28.0134	126	3.395	1.26985	3.26557	88.136
Hexatriacontane	$C_{36} H_{74}$	507	872	0.47	13.91529	4.24904	288.462
Octacosane	$C_{28} H_{58}$	394.8	832	0.727	11.30955	4.16680	252.655
Eicosane	$C_{20} H_{42}$	282.5475	768	1.07	8.40357	4.20929	248.984
Hexadecane	$C_{16} H_{34}$	226.41	723	1.4	7.06791	4.07765	245.032
n-Decane	$C_{10} H_{22}$	142.285	618	2.11	4.6627	3.8384	243.87

## Evaluation of models

The following statistical parameters, namely root mean square error (*RMSE*), standard deviation (*SD*), and coefficient of determination (*R*^*2*^) were used in this survey to evaluate the performance of models:

10 $$RMSE = \sqrt {\frac{1}{Z}\sum\limits_{i = 1}^{Z} {\left( {NS_{i,\exp } - NS_{i,pred} } \right)}^{2} }$$

11 $$R^{2} = 1 - \frac{{\sum\limits_{i = 1}^{Z} {(NS_{i,\exp } - NS_{i,pred} )^{2} } }}{{\sum\limits_{i = 1}^{Z} {(NS_{i,\exp } - \overline{{NS_{\exp } }} )^{2} } }}$$

12 $$SD = \sqrt {\frac{1}{Z - 1}\sum\limits_{i = 1}^{Z} {\left( {\frac{{NS_{i,\exp } - NS_{i,pred} }}{{NS_{i,\exp } }}} \right)}^{2} }$$

where *Z*, *NS*_*i,exp*_, and *NS*_*i,pred*_ are the count of data, experimental N_2_ solubility, and predicted N_2_ solubility in normal alkanes, respectively.

On the other hand, the following graphical tools were utilized simultaneously to evaluate the performance of the ML models:

Cross plot: The most well-known graphical analysis in which the predicted values are plotted against the measured values and the accuracy of the models is evaluated by examining the proximity of the data points to the unit slop line.

Trend plot: This plot helps to check the validity of the model by sketching both real data and the model's estimation versus the specific property or data index.

Error distribution plot: The error (measured value − predicted value) is plotted against the real data to assess the scatter of data around the zero-error line and to explore the possible error trend.

Histogram plot of errors: This graph shows how the errors from the model are distributed. This statistical tool indicates the discrepancy between the measured and predicted values, in which a normal distribution centered at zero error is expected for a good model.

## Results and discussion

### Model optimization and tuning

To find the best model in each aforementioned algorithm, a routine procedure has been done to find the hyperparameters and the other functional features of each model. Since these models have been implemented in python, different libraries including scikit-learn for k-NN and Random forest^110^, xgboost for XGBoost, lightgbm for LightGBM^98^, and catboost^99^ for Catboost have been employed in this study. In each of these involves some parameters that should be set by user or they can be work on default mode. To find the best model state in each of algorithms, a wide range of selective parameters have been selected and the best model based on the training and test data RMSE has been chosen. The search space and the final arrangements of model are provided in Table 6.

**Table 6:** Models' tuning search space and selected model based on RMSE.

Model	Search space	No. of tuning models	Selected model
k-NN	k = [1, 20], weights = [Distance, Uniform], algorithm = [Auto, Ball tree, KD tree, Brute], leaf size = [10,100, step = 10], distance = [Manhattan, Euclidean]	3200	k = 2, weights = Uniform, algorithm = Auto, leaf size = 10, distance = Euclidean
Random forest	Tree numbers = [10,200, step = 10], Criterion = [MSE, MAE], Max features = [Auto, Sqrt, log2]	120	Tree numbers = 120, Criterion = mse, Max features = Auto
XGBoost	Max depth = [3,10, step = 1], Subsample = [0.8, 0.9, 1], Booster = [gbtree, gblinear, dart], Learning rate = [0.01, 0.05, 0.1]	216	Max depth = 6, Subsample = 0.8, Booster = dart, Learning rate = 0.05
LightGBM	Number of leaves = [5, 10, 20, 30, 40, 50], Learning rate = [0.01, 0.05, 0.1, 0.2], Max depth = [6,10, step = 1]	120	Number of leaves = 50, Learning rate = 0.2, Max depth = 10
Catboost	Tree depth = [6,10, step = 1], Learning rate = [0.01, 0.05, 0.1], Loss function = [RMSE, MAE]	30	Tree depth = 10, Learning rate = 0.1 Loss function = MAE

### Statistics and performance metrics of the models

The model’s precision in predicting N_2_ solubility in normal alkanes was assessed statistically based on several statistical criteria including RMSE, R^2^, and SD. Table 7 reports the calculated values of these statistical factors for the training subset, testing subset, and the entire dataset of all ML models. The possibility of overtraining is completely rejected given that no meaningful difference was seen between the testing and training subsets for all models. Based on Table 7, the CatBoost model has the lowest prediction errors among the developed ML models with RMSE values of 0.0125, 0.0213, and 0.0147 for the training subset, testing subset, and the entire dataset, respectively. Also, the overall R^2^ of 0.9943 for the CatBoost model is higher than other models and has a lower SD, indicating a better fit for this model to the experimental data. Moreover, random forest, XGBoost, LightGBM, and k-NN models are categorized after the CatBoost model in terms of good performance, respectively.

**Table 7:** ML models’ statistics and performance metrics.

Model		RMSE	SD	R^2^
k-NN	Total	0.0276	0.4632	0.9802
	Train	0.0259	0.4799	0.9825
	Test	0.0336	0.3901	0.9716
Random forest	Total	0.0208	0.2361	0.9886
	Train	0.0170	0.1820	0.9931
	Test	0.0319	0.3826	0.9760
XGBoost	Total	0.0241	0.8669	0.9859
	Train	0.0219	0.9005	0.9884
	Test	0.0316	0.7181	0.9767
LightGBM	Total	0.0295	0.7002	0.9790
	Train	0.0276	0.6415	0.9801
	Test	0.0328	0.8981	0.9729
CatBoost	Total	0.0147	0.1739	0.9943
	Train	0.0125	0.1219	0.9960
	Test	0.0213	0.3032	0.9887

As mentioned earlier, several EOSs have been used comparatively with the ML models to estimate N_2_ solubility in normal alkanes. Hence, the solubilities of N_2_ in several normal alkanes namely Hexadecane, Eicosane, Octacosane, and hexatriacontane, which experimental values have been reported in the literature^29,90^, are estimated utilizing ML models and EOSs. Tables 8, 9, 10 and 11 represented the N_2_ solubility data and predictions of EOSs and ML models along with RMSE values for each of them. As can be seen, the CatBoost model provides the best estimates among the ML models and EOSs for the N_2_ solubility in all considered normal alkanes. ZJ EOS also had precise estimations for solubility values and outperformed other EOSs. On the other hand, as shown in Table 3, the Péneloux-type volume translation (*c*) has been used in the PR and SRK EOSs for the sake of investigation. Based on our studies, Péneloux-type volume translation does not have any effect on the obtained solubility values^111,112^.

**Table 8:** Estimations of different EOSs and ML models for N_2_ solubility in Hexadecane.

Temperature (K)	Pressure (MPa)	Experiment (mole fraction)	PR	SRK	RK	ZJ	PCSAFT	k-NN	Random forest	CatBoost	XGBoost	LightGBM
323.15	4.9	0.073	0.073892	0.071625	0.144902	0.063415	0.0768	0.037175	0.071356	0.073036	0.082158	0.070108
323.15	9.8	0.135	0.136743	0.132524	0.261235	0.117323	0.1386	0.104	0.131207	0.134779	0.136789	0.123276
323.15	19.6	0.223	0.237912	0.230084	0.433672	0.203614	0.2308	0.231	0.218454	0.222653	0.214433	0.218651
323.15	29.4	0.282	0.315963	0.304564	0.550651	0.269623	0.2960	0.294	0.291091	0.282016	0.291839	0.280639
323.15	39.2	0.326	0.378184	0.363228	0.640202	0.321973	0.3612	0.345	0.333536	0.325985	0.328946	0.322025
323.15	49	0.36	0.429033	0.410621	0.705706	0.364692	0.4264	0.3865	0.37509	0.360008	0.367519	0.349678
373.15	4.9	0.078	0.074263	0.073492	0.146725	0.069883	0.0892	0.039755	0.075178	0.078577	0.083729	0.072468
373.15	9.8	0.142	0.138759	0.13687	0.266309	0.130523	0.1620	0.11	0.140184	0.141646	0.140065	0.139086
373.15	19.6	0.239	0.245062	0.240172	0.447042	0.230063	0.2726	0.246	0.234459	0.238616	0.224191	0.24045
373.15	29.4	0.306	0.328896	0.320386	0.575301	0.308027	0.3521	0.3185	0.310965	0.304803	0.303363	0.306795
373.15	39.2	0.364	0.396591	0.384215	0.670128	0.370614	0.4118	0.3815	0.366843	0.364023	0.351979	0.352143
373.15	49	0.413	0.452314	0.436068	0.743035	0.421921	0.4715	0.4365	0.420793	0.41301	0.395695	0.387751
423.15	4.9	0.093	0.077963	0.078507	0.152567	0.077978	0.1020	0.07395	0.089375	0.087169	0.093703	0.086517
423.15	9.8	0.158	0.146119	0.146234	0.277521	0.146155	0.1851	0.1556	0.158329	0.150375	0.171655	0.161286
423.15	19.6	0.253	0.259183	0.256632	0.467131	0.25905	0.3116	0.27415	0.261208	0.252872	0.266006	0.26376
423.15	29.4	0.331	0.348808	0.342327	0.6028	0.348188	0.3902	0.3185	0.346806	0.331465	0.349459	0.341662
423.15	39.2	0.399	0.421327	0.410442	0.704483	0.420007	0.4702	0.3815	0.418967	0.398995	0.417612	0.403085
423.15	49	0.46	0.481033	0.465681	0.784954	0.47891	0.5495	0.4365	0.48882	0.459886	0.474527	0.46766
473.15	4.9	0.1015	0.084643	0.086472	0.161361	0.085461	0.1164	0.09635	0.097778	0.100989	0.096573	0.095005
473.15	9.8	0.176	0.158538	0.160499	0.293945	0.160388	0.2107	0.1726	0.176568	0.172191	0.177822	0.163749
473.15	19.6	0.287	0.280484	0.279759	0.494149	0.284606	0.3528	0.29115	0.299906	0.289682	0.315014	0.308673
473.15	29.4	0.377	0.376499	0.371171	0.637714	0.38278	0.4538	0.3599	0.38452	0.377019	0.378863	0.368444
473.15	39.2	0.455	0.453705	0.443122	0.747637	0.461885	0.5282	0.4855	0.483065	0.45509	0.469246	0.47425
473.15	49	0.527	0.516916	0.501016	0.843771	0.526735	0.5848	0.56	0.544546	0.526918	0.518121	0.527232
		RMSE	0.024546	0.017951	0.236762	0.012009	0.04857	0.021663	0.011942	0.002204	0.011869	0.010353

**Table 9:** Estimations of different EOSs and ML models for N_2_ solubility in Eicosane.

Temperature (K)	Pressure (MPa)	Experiment (mole fraction)	PR	SRK	RK	ZJ	PCSAFT	k-NN	Random forest	CatBoost	XGBoost	LightGBM
323.2	4.49	0.061	0.069247	0.072369	0.156472	0.0647	0.0978	0.06965	0.069154	0.05889	0.069354	0.064474
323.2	5.13	0.0689	0.078258	0.081793	0.176046	0.073128	0.1099	0.06965	0.069809	0.068936	0.081651	0.070776
323.2	5.25	0.0704	0.079926	0.083537	0.179648	0.074688	0.1121	0.06965	0.070881	0.070321	0.081651	0.071701
323.2	7.54	0.0967	0.110494	0.115507	0.244533	0.103277	0.1522	0.08355	0.096815	0.096834	0.107496	0.097726
323.2	10.61	0.1292	0.148044	0.154761	0.321219	0.138373	0.1993	0.11295	0.133487	0.129172	0.143991	0.137048
323.2	11.9	0.1413	0.162768	0.170142	0.350366	0.152123	0.2171	0.15405	0.155163	0.139204	0.153521	0.14747
323.2	16.22	0.1789	0.208111	0.217434	0.436792	0.194401	0.2700	0.18275	0.186544	0.177966	0.192096	0.19271
323.2	17.23	0.1866	0.217914	0.227641	0.454814	0.203529	0.2811	0.18275	0.190868	0.18667	0.199734	0.200127
373.2	4.03	0.0629	0.062276	0.065174	0.140174	0.062646	0.0999	0.0697	0.06235	0.058326	0.055584	0.065675
373.2	4.61	0.0715	0.070622	0.073885	0.158237	0.071043	0.1126	0.0702	0.071369	0.071417	0.069449	0.072104
373.2	8.33	0.1199	0.120877	0.126194	0.263387	0.12159	0.1859	0.10395	0.132528	0.127787	0.154828	0.125944
373.2	9.74	0.1364	0.138558	0.144536	0.298913	0.13936	0.2105	0.15015	0.137541	0.137141	0.143446	0.136792
373.2	12.1	0.1639	0.166634	0.173592	0.353769	0.167558	0.2483	0.15015	0.165703	0.16413	0.168247	0.165751
373.2	14.61	0.1905	0.194577	0.202417	0.406485	0.195592	0.2844	0.1772	0.193454	0.18934	0.193176	0.201639
423.2	3.83	0.0679	0.061614	0.064751	0.136393	0.065181	0.1058	0.08045	0.065761	0.067853	0.060686	0.059676
423.2	5.38	0.093	0.084663	0.088807	0.185062	0.089567	0.1428	0.08045	0.094388	0.093002	0.093197	0.09094
423.2	7.76	0.1278	0.118145	0.123589	0.253441	0.124987	0.1942	0.13615	0.125303	0.125503	0.134516	0.126349
423.2	8.89	0.1445	0.133285	0.139252	0.283474	0.140998	0.2167	0.13615	0.151786	0.146367	0.170064	0.148176
423.2	11.09	0.1728	0.161462	0.168293	0.337927	0.170784	0.2570	0.15865	0.181252	0.172828	0.180376	0.190832
423.2	14.24	0.2121	0.199048	0.20681	0.407718	0.210489	0.3083	0.19245	0.216864	0.212101	0.231485	0.215076
		RMSE	0.013682	0.017248	0.16285	0.006777	0.06872	0.011408	0.005864	0.002276	0.013652	0.007367

**Table 10:** Estimations of different EOSs and ML models for N_2_ solubility in Octacosane.

Temperature (K)	Pressure (MPa)	Experiment (mole fraction)	PR	SRK	RK	ZJ	PCSAFT	k-NN	Random forest	CatBoost	XGBoost	LightGBM
348.2	4.3	0.0726	0.066536	0.078483	0.187781	0.075533	0.1051	0.0794	0.070957	0.072598	0.072464	0.079949
348.2	6.93	0.1108	0.102759	0.12115	0.282369	0.116653	0.1576	0.1221	0.103017	0.110783	0.112164	0.109869
348.2	8.04	0.1245	0.117152	0.138085	0.318477	0.132984	0.1776	0.1221	0.128687	0.125423	0.138509	0.119371
348.2	8.7	0.1334	0.125475	0.147873	0.338976	0.142424	0.1890	0.1221	0.130848	0.133295	0.14151	0.135998
348.2	13.7	0.1909	0.183373	0.215831	0.474004	0.208019	0.2641	0.19045	0.187373	0.190084	0.179635	0.193985
348.2	16.47	0.2181	0.211983	0.249312	0.535941	0.240375	0.2987	0.19045	0.204173	0.214211	0.179635	0.215489
373.2	4.87	0.0862	0.074604	0.086625	0.205827	0.086208	0.1248	0.20945	0.082122	0.086196	0.211254	0.094801
373.2	5.63	0.0988	0.085242	0.098939	0.233281	0.098494	0.1413	0.0925	0.096927	0.099156	0.088713	0.102459
373.2	9.08	0.1466	0.130556	0.151272	0.345099	0.150787	0.2081	0.0925	0.141822	0.146078	0.096741	0.150893
373.2	10.89	0.1698	0.152539	0.176585	0.39642	0.176125	0.2388	0.1582	0.172329	0.169884	0.14843	0.181823
373.2	14.18	0.2071	0.189699	0.21925	0.478967	0.2189	0.2881	0.1582	0.202881	0.208983	0.169716	0.213385
373.2	16.1	0.2289	0.209871	0.24234	0.521621	0.242087	0.3136	0.218	0.225081	0.229179	0.206457	0.238142
423.2	4.46	0.0896	0.070605	0.080209	0.188867	0.083902	0.1290	0.218	0.086009	0.089635	0.219019	0.093516
423.2	5.11	0.101	0.080124	0.090957	0.212741	0.095202	0.1451	0.0953	0.0995	0.100356	0.076733	0.107104
423.2	9.31	0.1689	0.137473	0.155382	0.349017	0.163206	0.2360	0.0953	0.166621	0.168771	0.095737	0.172708
423.2	11.07	0.1951	0.159546	0.180026	0.39815	0.189337	0.2685	0.13495	0.186831	0.200133	0.174195	0.210017
423.2	13.94	0.232	0.193334	0.217579	0.469966	0.229281	0.3158	0.16935	0.232566	0.243504	0.188552	0.234811
423.2	16.01	0.2578	0.216144	0.242814	0.516219	0.256208	0.3462	0.188	0.257379	0.261813	0.24326	0.259564
		RMSE	0.021252	0.014062	0.211599	0.009122	0.0644	0.055889	0.005081	0.00329	0.051468	0.006583

**Table 11:** Estimations of different EOSs and ML models for N_2_ solubility in Hexatriacontane.

Temperature (K)	Pressure (MPa)	Experiment (mole fraction)	PR	SRK	RK	ZJ	PCSAFT	k-NN	Random forest	CatBoost	XGBoost	LightGBM
373.2	5.3	0.1054	0.100115	0.119624	0.27005	0.1091	0.1122	0.1191	0.086086	0.107791	0.098838	0.098058
373.2	6.1	0.1197	0.113623	0.135666	0.303165	0.12391	0.1265	0.15655	0.110326	0.119716	0.110251	0.112788
373.2	11.1	0.1934	0.190101	0.226027	0.476773	0.208135	0.2043	0.15655	0.183293	0.192674	0.183441	0.183233
373.2	12.23	0.2089	0.205684	0.244338	0.509363	0.225374	0.2195	0.2281	0.178168	0.220062	0.197588	0.185332
373.2	16.81	0.2628	0.263397	0.311838	0.622316	0.289448	0.2740	0.26885	0.249988	0.262435	0.247149	0.248294
373.2	17.99	0.2749	0.276987	0.327659	0.647204	0.304591	0.2880	0.26885	0.260137	0.275036	0.265712	0.263283
423.2	5.28	0.1185	0.100832	0.117151	0.264578	0.111689	0.1288	0.16125	0.110294	0.11853	0.103108	0.108029
423.2	5.56	0.124	0.105676	0.122727	0.276162	0.117085	0.1347	0.16125	0.111694	0.124989	0.115516	0.116324
423.2	10.22	0.204	0.179957	0.207662	0.442046	0.200167	0.2203	0.16125	0.188917	0.201519	0.18712	0.205322
423.2	11.71	0.2263	0.201423	0.232003	0.48608	0.22429	0.2439	0.23935	0.195252	0.206822	0.198636	0.210017
423.2	15.21	0.2747	0.248073	0.284586	0.576178	0.27689	0.2933	0.28585	0.259206	0.274785	0.261637	0.255037
423.2	17.11	0.297	0.27139	0.310707	0.618474	0.303271	0.3172	0.28585	0.284358	0.296956	0.281106	0.26342
		RMSE	0.016584	0.026301	0.268031	0.01375	0.01345	0.02709	0.017559	0.006567	0.014350	0.015957

### Graphical analysis of the models

**Figure 3:**
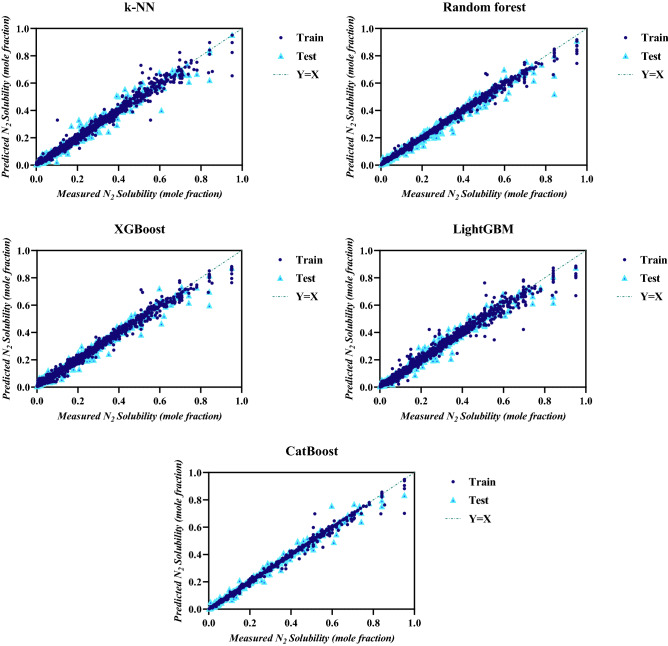
Cross plots of experiments vs predictions for the ML models.

In the next step, the evaluation of the ML models is performed by graphical analysis. First, cross plots of the experimental N_2_ solubility data versus predicted values by the ML models for the training and testing stages are presented in Fig. 3. All five ML models performed well in both training and testing stages and most of the data points are accumulated around the X = Y line, although the scatter of points is much less for the CatBoost model and is more concentrated around the X = Y line, indicating the excellent performance of this model in estimating N_2_ solubility in normal alkanes.

**Figure 4:**
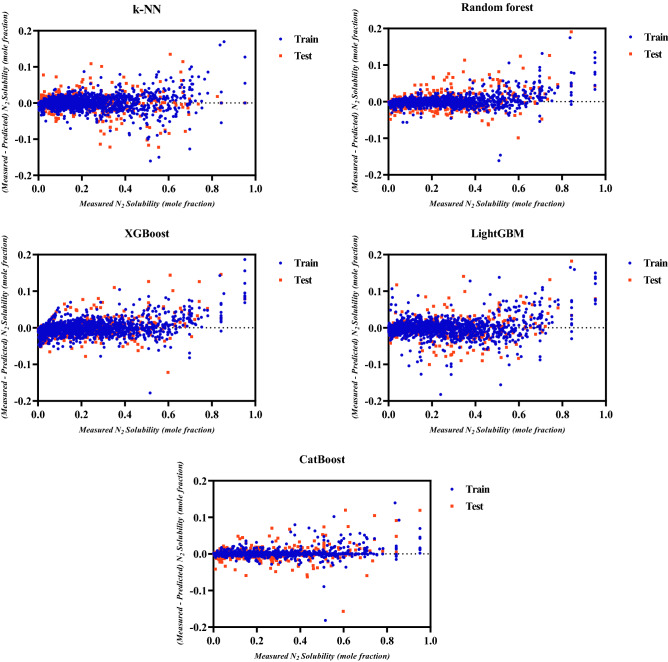
Prediction error distributions of ML models.

Next, the distributions of the N_2_ solubility prediction errors (measured—predicted) utilizing the ML models versus the experimental data are shown in Fig. 4. High concentrations of near-zero error points for a predictive tool indicate a better performance of that predictive tool in predicting N_2_ solubility in normal alkanes. Again, the CatBoost model resulted in near-zero errors, verifying its accuracy and reliability. However, other ML models especially random forest shows good predictions with low errors for the N_2_ solubility in normal alkanes.

**Figure 5:**
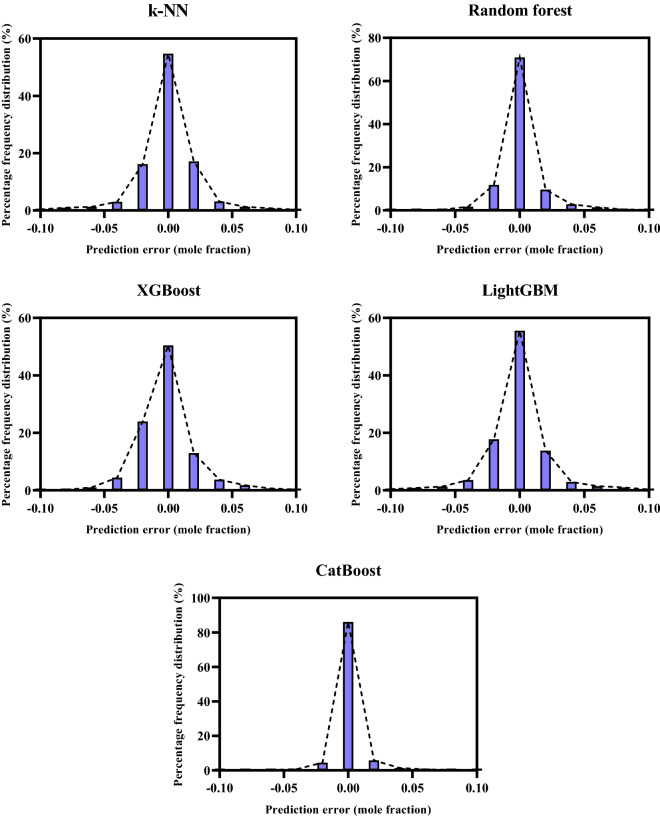
Histograms of errors for the ML models.

The next step of the graphical assessment of introduced ML models for the prediction of N_2_ solubility in normal alkanes is related to the frequency of errors. Figure 5 depicts the histograms of errors corresponding to the proposed ML models in this work. As it is clear, the symmetric distributions are seen in the histogram graphs of all ML models. Also, the bursts of growing at the zero-error value for all developed models confirm the superb match between estimated and experimental data of N_2_ solubility in normal alkanes. However, the percentage frequency of errors at the zero-error value is about 85% for the CatBoost model and it is much higher than other ML models indicating the high credit of this model in estimating N_2_ solubility in normal alkanes.

However, all the models used in this study show satisfactory performances. As it is obvious from the statistical and graphical analyses, the CatBoost model shows the best performance among the implemented ML models. The performance of a model depends on many factors, such as the case of study and the structure of the dataset, and this superiority in performance for this model stems from two main reasons. The first one is the structure of the dataset used in this work, based on the shape of the dataset, there are many instances that have equal values in the n-1 feature and their only difference is in one feature. This feature enables the tree-based models to do a better splitting operation and finally brings higher accuracy. Secondly, Catboost models use symmetric trees and it helps to have a faster inference. Also, its boosting schemes are the main reason which avoids overfitting and increases the model quality after the training process. Finally, it should be noted that these advantages for Catboost strongly depend on the dataset and it cannot be generalized to all problems.

### Pressure and temperature trend analysis

**Figure 6:**
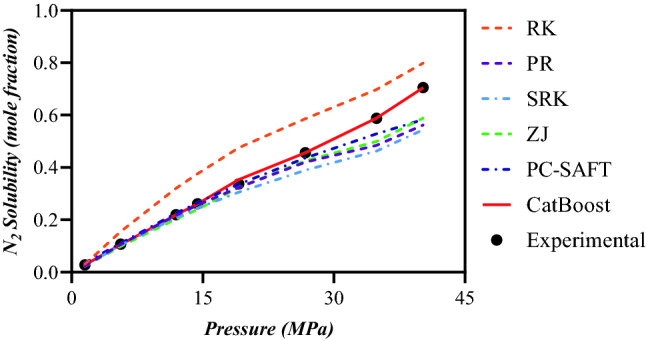
Pressure trend analysis of N_2_ solubility based on the results of various EOSs and Catboost ML model for n-Decane at T = 503 K.

As the final assessment step, various visual evaluations were executed to appraise the CatBoost model's capability in various N_2_ solubility in hydrocarbons systems. Figure 6 represents the effect of pressure on N_2_ solubility for n-Decane system at the temperature of 503 K. Figure 6 shows N_2_ solubilities estimated by the CatBoost model for this case, as well as the values determined by the EOSs along with the literature experimental results^87^. The mismatch between standard EOSs estimations and actual experimental data is quite significant at high temperatures. As seen in this figure, the CatBoost model predicts experimental data quite well. Based on expectations, the solubility of N_2_ in n-Decane rises as the pressure increases. Meanwhile, the EOSs overestimate or underestimate the N_2_ solubility ‘growth when pressure rises, while the CatBoost model strictly traces the trend.

**Figure 7:**
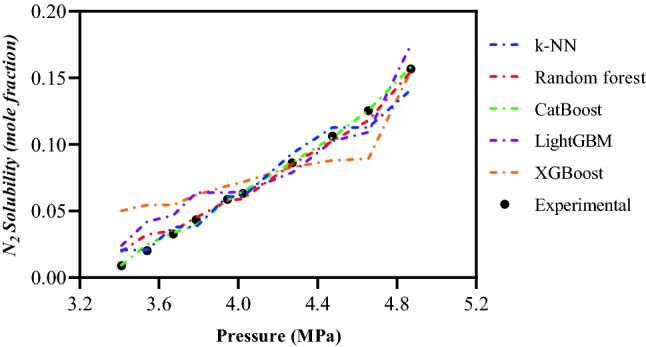
Pressure trend analysis of N_2_ solubility based on the results of implemented ML models for Methane at T = 180 K.

The predictions of CatBoost and other proposed ML models for N_2_ solubility data in a light hydrocarbon (methane)^61^ under various operation conditions at a constant temperature of 180 K are provided in Fig. 7. All the intelligent models follow the trend well, and show a positive trend in N_2_ solubility as pressure increases. The CatBoost model, as shown in this figure, accurately recognizes data patterns and provides excellent estimations in all pressures.

**Figure 8:**
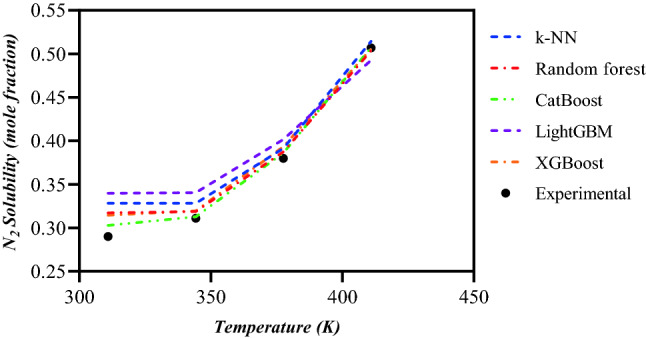
Temperature trend analysis of N_2_ solubility based on the results of implemented ML models for n-hexane at P = 27.57 MPa.

Finally, a similar trend analysis performed to investigate the performance of different ML models at various temperature states to estimate the N_2_ solubility in n-hexane at the constant pressure of 27.57 MPa^74^. Based on Fig. 8, similar to the previous case, a satisfactory trend capturing is observed in all the intelligent models. However, the Catboost model provides more accurate predictions. Also, the figure indicates an increase in N_2_ solubility as temperature rises.

### Sensitivity analysis

**Figure 9:**
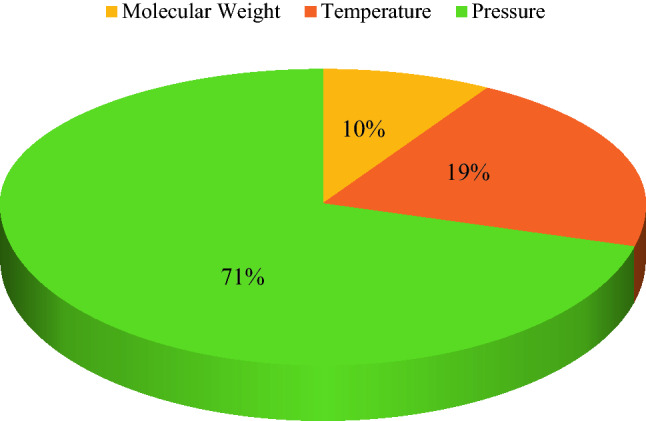
Relevancy factor analysis.

Utilizing the CatBoost model as the best-developed model in the current study, a sensitivity analysis was performed. To this end, the relevancy factor (*r*)^113^ was calculated for each input parameter using the following equation, with the knowledge that the higher the *r*-value, the greater impact on the model's output. It should also be noted that the positive *r*-value for a parameter indicates its direct effect on the output of the model and vice versa^114^.

13 $$r(I_{i} ,NS) = \frac{{\sum\limits_{j = 1}^{n} {\left( {I_{i,j} - I_{m,i} } \right)\left( {NS_{j} - NS_{m} } \right)} }}{{\left( {\sum\limits_{j = 1}^{n} {\left( {I_{i,j} - I_{m,i} } \right)^{2} \sum\limits_{j = 1}^{n} {\left( {NS_{j} - NS_{m} } \right)^{2} } } } \right)^{0.5} }}$$

where *I*_*i,j*_ represents the *j*th value of the *i*th input variable (*i* is molecular weight of normal alkanes, pressure, and temperature); *I*_*m,i*_ shows mean value of the *i*th input; *NS*_*m*_ and *NS*_*j*_ denote the mean value and the *j*th value of predicted N_2_ solubility in normal alkanes, respectively. The outcomes of the relevancy factor analysis are depicted in Fig. 9. According to Fig. 9, all input parameters, namely temperature, pressure, and molecular weight of normal alkanes have a positive effect on N_2_ solubility in normal alkanes. The results reveal that the pressure has the greatest impact on N_2_ solubilities in normal alkanes and the N_2_ solubility increases with increasing the molecular weight of normal alkanes. Based on Henry's law, the amount of dissolved gas in a liquid is proportional to its partial pressure in equilibrium with that liquid. When the gas is at a higher pressure, its molecules collide more with each other and with the liquid's surface. As the molecules collide more with the surface of the liquid, they can squeeze between the liquid molecules and thus become a part of the solution^115,116^. On the other hand, the sensitivity analysis overall shows that the solubility of N_2_ in normal alkanes increases when the temperature increases. This shows the reverse order solubility phenomenon that is the opposite of what commonly happens for a binary mixture of a supercritical component and a subcritical component^73,81^. The reason for this may be due to the repulsive nature of N_2_–N_2_ interaction. The N_2_–N_2_ repulsive force decreases with an increase in temperature, which results in increased solubility of N_2_ at higher temperatures. However, increasing the solubility of N_2_ with an increase in temperature may not be true for all normal alkanes and literature survey shows that the N_2_ solubility in methane and ethane decreases with increasing temperature^117^. Normal alkanes are nonpolar, as they contain nothing but C–C and C–H bonds. N_2_ is also a nonpolar molecule and nonpolar substances tend to dissolve in nonpolar solvents such as normal alkanes. The molecular weight of the normal alkanes is mainly increased by adding C–C and C–H bonds. The obvious consequence of this is that the N_2_ solubility increases as the number or length of the nonpolar chains increases.

## Conclusions

In the present work, N_2_ solubility in normal alkanes (nC_1_ to nC_36_) was modeled using five representative ML models namely CatBoost, k-NN, LightGBM, random forest, and XGBoost by utilizing a large N_2_ solubility databank in a wide range of operating temperature (91.21–703.4 K) and pressure (0.0212–69.12 MPa). Also, five EOSs namely RK, SRK, ZJ, PR, and PC-SAFT were used comparatively with the ML models to estimate N_2_ solubility in normal alkanes. The developed CatBoost model was superior to all of ML models and EOSs with an overall RMSE of 0.0147 and R^2^ of 0.9943. Moreover, Random Forest, XGBoost, LightGBM, and k-NN models were ranked after the CatBoost model in terms of good performance, respectively. Furthermore, ZJ EOS showed the best performance among the EOSs. Finally, the results of relevancy factor analysis indicated that all input variables to the models, namely temperature, pressure, and molecular weight of normal alkanes have a positive effect on N_2_ solubilities in normal alkanes and pressure has the greatest effect among these input variables. The solubility of N_2_ increases with increasing the molecular weight of normal alkanes.

## Abbreviations

CARTs: Classification and regression trees
CNN: Convolutional neural network
EOR: Enhanced oil recovery
EOS: Equation of state
exp: Experimental
k-NN: K-nearest neighbors
ML: Machine learning
Mw: Molecular weight
NS: Nitrogen solubility
PC-SAFT: Perturbed-Chain Statistical Associating Fluid Theory
PR: Peng-Robinson EOS
pred: Predicted
RMSE: Root mean square error
RK: Redlich-Kwong EOS
SAFT: Statistical associating fluid theory
SRK: Soave–Redlich–Kwong EOS
SD: Standard deviation
SW: Schmidt-Wenzel EOS
VLE: Vapor–liquid equilibria
XGBoost: EXtreme Gradient Boosting

## Subscript and superscript

N_2_: Nitrogen
R^2^: Coefficient of determination
P_c_: Critical pressure
T_c_: Critical temperature
